# Retention of a Copper T380A Intrauterine Device for 29 Years in a 64-Year-Old Female With Postmenopausal Bleeding

**DOI:** 10.7759/cureus.82444

**Published:** 2025-04-17

**Authors:** Mia Glickman, Jessica Cohen, Parvathi Perumareddi

**Affiliations:** 1 Family Medicine, Florida Atlantic University Charles E. Schmidt College of Medicine, Boca Raton, USA; 2 Obstetrics and Gynecology, Nova Southeastern University Dr. Kiran C. Patel College of Osteopathic Medicine, Fort Lauderdale, USA

**Keywords:** contraception literacy, copper intrauterine device, evaluation of postmenopausal bleeding, healthcare literacy, postmenopausal bleeding

## Abstract

Intrauterine devices (IUDs) are widely utilized, long-acting reversible contraceptives with established guidelines for timely removal. Prolonged IUD retention into the postmenopausal period is an uncommon phenomenon but may have various effects on the endometrium. We present the case of a 64-year-old female who presented with postmenopausal bleeding (PMB) and was incidentally found to have a copper T380A IUD retained in utero for 29 years. The IUD was removed without complications, and her bleeding resolved following removal. However, endometrial biopsy revealed hyperplasia without atypia. Although retention of the IUD posed no major complications for this patient, it is unknown whether its presence contributed to her presentation of PMB. Potential adverse effects and complications of prolonged IUD use are also important to consider and are discussed therein. A brief review of case reports highlighting the outcomes of prolonged IUD retention is also provided.

## Introduction

Intrauterine devices (IUDs) are a common method of long-acting reversible contraception that have a high rate of efficacy, safety, and patient satisfaction [1]. IUDs come in both hormonal (progestin-containing) and non-hormonal (copper-containing) modalities. The copper T380A (ParaGard) IUD was approved by the U.S. Federal Drug Administration (FDA) in 1984. It was originally approved for up to four years of use, but as of March 2024, it can be used for up to 10 years [2]. Although there are some studies that demonstrate effective contraception for up to 12 years of use [3], this duration is not yet approved by the FDA. IUD removal can occur in an outpatient setting as a brief and safe procedure. IUD retention past the intended duration of use can lead to several complications, including intraabdominal infection, uterine (or adjacent organ) perforation, and menstrual cycle disruption, including both heavy menstrual bleeding and amenorrhea [2]. However, since prolonged IUD retention is an uncommon phenomenon, there are minimal data to reflect the incidence of these complications. This case discusses the course of a ParaGard IUD retained for 29 years in a 64-year-old G2P2 female from Colombia.

## Case presentation

A 64-year-old Spanish-speaking female with no significant past medical history presented to a free community clinic for follow-up one month after a transvaginal ultrasound (TVUS) was performed for the evaluation of postmenopausal bleeding (PMB). At the initial visit, the patient reported intermittent vaginal bleeding for five months. She initially only noted scant blood on the toilet paper when wiping but became concerned when she began having bleeding similar to that of a menstrual cycle. She denied any passage of clots. These bleeding episodes occurred approximately twice a month. A complete blood count showed a stable hemoglobin of 13.5 g/dL. A complete pelvic examination was deferred by the patient, but external palpation of the uterus revealed no tenderness or enlargement. Gynecologic history was notable for menarche at age 11 and menopause at age 45. A Pap smear performed in March 2024 revealed no intraepithelial malignancy. Per chart review, there was no mention of visible IUD strings or discussion of IUD retention at this time. She denied any family history of gynecologic cancer or personal use of hormone replacement therapy. She has never been diagnosed with a sexually transmitted infection. She takes no medications and denies any pelvic trauma. There was also no history of surgeries. The patient’s BMI was 31.23 kg/m^2^.

**Figure 1:**
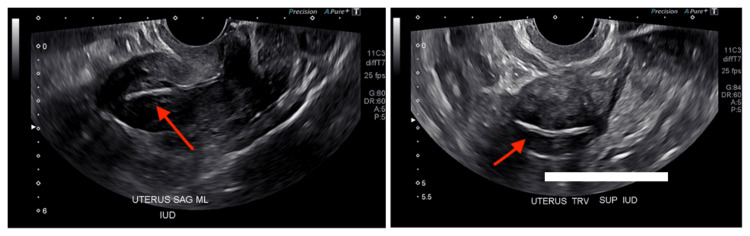
Sagittal and transverse transvaginal ultrasound images revealing the presence of the retained intrauterine device

The diagnostic TVUS, ordered as a part of the workup for PMB, revealed an endometrial wall thickness of 14 mm; a measurement of >4 mm is generally an indication for an endometrial biopsy, as it indicates high risk for malignancy [4]. Incidentally, the ultrasound report revealed the presence of an IUD, which was not disclosed in prior patient interviews with the gynecology provider (Figure 1). The patient did not report any symptoms that may have indicated an IUD-related complication aside from the PMB.

At a follow-up visit to discuss her ultrasound findings, the patient was asked about the details of her IUD placement. She provided a card, which displayed a record of placement on August 31, 1995. The patient stated that she had the IUD placed in the postpartum period following her second vaginal delivery while living in Colombia. She revealed that she was never able to have the IUD removed due to limited healthcare access. She moved to the United States in 1999 and is undocumented and uninsured. The patient consented to a pelvic examination, and the IUD strings were visible upon speculum placement. The IUD was removed without complications and sent to the lab for cultures, which later revealed the growth of *Citrobacter freundii *(sensitive to cefepime, ceftriaxone, ciprofloxacin, gentamicin, levofloxacin, meropenem, and trimethoprim-sulfamethoxazole). An endometrial biopsy was also performed, which later showed endometrial hyperplasia without atypia.

Following the removal of the IUD, the patient has remained asymptomatic, and her vaginal bleeding has resolved. A subsequent TVUS was performed, which revealed a complex cystic mass in the endometrial cavity. Given her elevated cancer risk and abnormal radiographic findings, the patient has expressed interest in hysterectomy. She was advised to apply for financial assistance funded by the clinic and is currently in the process of doing so. Of note, the patient is a fluent Spanish speaker with limited English proficiency, and all communications were completed through a remote language translator service.

## Discussion

This case of prolonged IUD retention highlights the need for further discussion on IUD subtypes and general guidelines for their duration of use. Additionally, it is important to consider potential complications, especially when usage extends beyond the recommended timeframe. Moreover, we examine a possible link between IUD retention, PMB, and endometrial changes.

IUDs come in two modalities: copper-containing and levonorgestrel-releasing [5]. The preferential use of one over another depends on the presence or absence of contraindications and indications for insertion. The copper-containing IUD is favored by those who prefer a non-hormonal approach to birth control. In contrast, the levonorgestrel-releasing IUD has broader applications for use beyond contraception, including the treatment of primary dysmenorrhea, heavy menstrual bleeding, and endometrial hyperplasia. The guidelines for IUD removal are dependent on device expiration (i.e., when the copper or progestin content is exhausted). Over the past two decades, newer device formulations have extended the duration of use. Current guidelines recommend removal after 10 years of use for the copper-containing IUD and three to eight years for the levonorgestrel-containing IUD, depending on the brand formulation [4].

According to the American College of Obstetricians and Gynecologists (ACOG), no clinical trials have been conducted to examine the risks of prolonged IUD retention in postmenopausal women [6]. Inadvertent IUD retention into the postmenopausal period is an uncommon phenomenon, given the structure of routine gynecologic practice. Several socioeconomic factors likely contributed to our patient's circumstance, including her undocumented status, limited English language proficiency, and lack of health insurance.

Based on the known complications of short-term use, proposed complications of prolonged retention include uterine perforation, pelvic infection, or abnormal uterine bleeding [7], such was the case in our patient. However, case reports exist in which prolonged IUD retention caused no symptoms, including a Lippes IUD retained for 50 years [8]. To date, no case reports have been published involving the retention of a ParaGard T380A copper-containing IUD for nearly three decades in a postmenopausal woman. In addition, few published case reports describing IUD retention in postmenopausal women were without complication. Regarding the ParaGard IUD, complicated removal due to string retraction and device arm fracture [9] have both been described. Pyometra and pelvic actinomyces have also been reported as adverse outcomes [10]. Another case report published in 2012 attributes PMB to the presence of a copper-7 IUD that had been retained for 30 years [11]. Importantly, the copper-7 IUD differs from the T380A, as it contains a much lower copper content, bears a different structure, and is not available in the United States [12]. Nonetheless, it is essential to consider whether or not retention of the T380A copper-containing IUD contributed to PMB in our patient. Copper-containing IUDs are classically associated with an increase in menstrual bleeding by virtue of their mechanism of triggering an inflammatory response. However, in our patient with device retention well past the intended duration, the copper content was likely exhausted, thus limiting a copper-induced inflammatory response. Therefore, any PMB associated with the presence of the IUD was likely a reaction to it as a foreign body. This patient’s bleeding resolved following the removal of the IUD, which supports the notion that the IUD was contributing to her symptoms to some degree.

Table 1 provides a summary of the literature on IUD-related complications and retention in postmenopausal women.

**Table 1:** Summary of literature on IUD-related complications and retention in postmenopausal women IUD, intrauterine device; G, gravida; P, para

Study	IUD Type	Key Findings	Complications	Comments
Aniulienė and Aniulis [8]	Lippes (polyethylene plastic)	A 74-year-old female, G2P2, who presented for evaluation of stress urinary incontinence and was found to have a Lippes IUD retained for 50 years in utero.	None	No major complications from prolonged IUD retention.
Koh [9]	Lippes (polyethylene plastic)	A 75-year-old female, G0P0, who was referred to urology for workup of microhematuria. A calculus was noted on imaging, and the patient underwent cystoscopy. Intraoperatively, a foreign device was found and later identified as a Lippes IUD.	Bladder perforation	Exact duration unknown due to lack of recollection of placement date.
Koh [9]	Copper T380A	A 59-year-old female, unknown G/P, presented requesting IUD removal. Manual extraction showed a fragmented device missing an arm. Transvaginal ultrasound revealed the fragmented segment embedded into the myometrium. Hysteroscopy was required for extraction.	Arm fracture, fragment embedment into uterine wall	Duration of device retention not certain but estimated to be 12 years.
Kriplani et al. [10]	Lippes (polyethylene plastic)	A 59-year-old female, G7P7, presented for the evaluation of malodorous vaginal discharge and intermittent fever. Pelvic examination showed strings belonging to a Lippes IUD. The patient had reportedly forgotten about the device, which was placed 22 years prior.	Pelvic actinomyces and pyometra	The authors noted a direct association between duration of IUD use and actinomycosis.
Wagner and Gimpelson [11]	Copper-7	A 64-year-old female, G1P0, presented to gynecology reporting period-like vaginal bleeding. An ultrasound revealed the presence of a copper-7 IUD that had been placed 30 years prior. Retrieval required hysteroscopic guidance.	Postmenopausal bleeding	This case is similar to ours, with key differences in IUD type (copper-7 vs. T380A) and retrieval method (hysteroscopic vs. manual).

Our patient presented with PMB and additional risk factors for endometrial carcinoma, including obesity and early menarche. However, her endometrial biopsy was negative, which may prompt us to consider the effect of copper-containing IUDs on the endometrial lining. Several studies have reported that there is a reduced risk of endometrial carcinoma in patients’ who have used a copper-containing IUD [13]. These findings are somewhat counterintuitive, given that copper IUDs promote an inflammatory reaction within the endometrium, and inflammation is known to drive carcinogenesis [14]. It is through the same mechanism, however, that there may be a protective benefit. Several proposed theories cite that the inflammatory response induces endometrial atrophy, which reduces cancer risk [15]. Progestin-containing IUDs are routinely used in postmenopausal women for the management of endometrial hyperplasia given their antagonistic effect on estrogen. Hence, there is room to question whether or not copper-containing IUDs could be used for similar indications given their proposed pro-atrophic effects. Copper-containing IUDs are more appropriate for women with contraindications to hormone therapy, such as those with hormone-sensitive breast cancer; hence, this constitutes a potential area for further research.

## Conclusions

IUDs are a safe and highly effective form of contraception with routine guidelines for insertion and removal. Given the sparsity of scenarios in which an IUD was retained into the postmenopausal period, it is difficult to establish causation and attribute PMB to this phenomenon. Prolonged IUD retention is intuitively associated with a significant risk of complications, but existing case reports have demonstrated scenarios in which there were no adverse effects of prolonged use and no complications of removal; such is the case for the patient presented here. Further research is needed to elucidate the long-term effects prolonged IUD use, and physicians should remain vigilant for potential IUD-related complications.
